# Percutaneous Radiofrequency Ablation for the Treatment of Peripheral Nerve Sheath Tumors: A Case Report and Review of the Literature

**DOI:** 10.7759/cureus.2534

**Published:** 2018-04-25

**Authors:** Oliver Mrowczynski, Christine Mau, Dan T Nguyen, Nabeel Sarwani, Elias Rizk, Kimberly Harbaugh

**Affiliations:** 1 Department of Neurosurgery, Penn State Hershey Medical Center, Hershey, USA; 2 Interventional Neuroradiology, Penn State Hershey Medical Center, Hershey, USA; 3 Radiology, Penn State Hershey Medical Center, Hershey, USA

**Keywords:** radiofrequency, peripheral nerve tumors, neurofibroma, schwannoma, review

## Abstract

Peripheral nerve sheath tumors (PNSTs) may arise sporadically or in the presence of genetic disorders, including neurofibromatosis (NF) types 1 and 2, schwannomatosis, and in patients with large genetic deletions involving the CDKN2A gene. Surgical resection is the treatment of choice for symptomatic PNSTs and offers patients a potential cure; however, pre-existing conditions or tumor location may limit a patient’s surgical options. Radiofrequency ablation (RFA) may provide an alternative therapeutic strategy for the treatment of selected PNSTs that are not amenable to surgical resection. Here, we present a case report of a 49-year-old patient with multiple neurofibromas who underwent RFA treatment of two symptomatic retroperitoneal neurofibromas and review previously reported cases of percutaneous treatment of PNSTs.

## Introduction

Although surgery is the current standard of care for the treatment of symptomatic peripheral nerve sheath tumors [1-3], comorbidities and tumor location may preclude surgery and, thus, alternative therapeutic approaches are necessary. Radiofrequency thermal ablation has become an increasingly utilized technique for cancer treatment due to its minimally invasive nature combined with the accuracy of current image-guided technologies [4]. Although radiofrequency ablation (RFA) has been studied in multiple cancer types [4], its use in the treatment of peripheral nerve sheath tumors (PNSTs) has been limited. Here, we present the first case of the RFA treatment of benign retroperitoneal neurofibromas and review experiences of the percutaneous treatment of peripheral nerve sheath tumors.

## Case presentation

**Figure 1:**
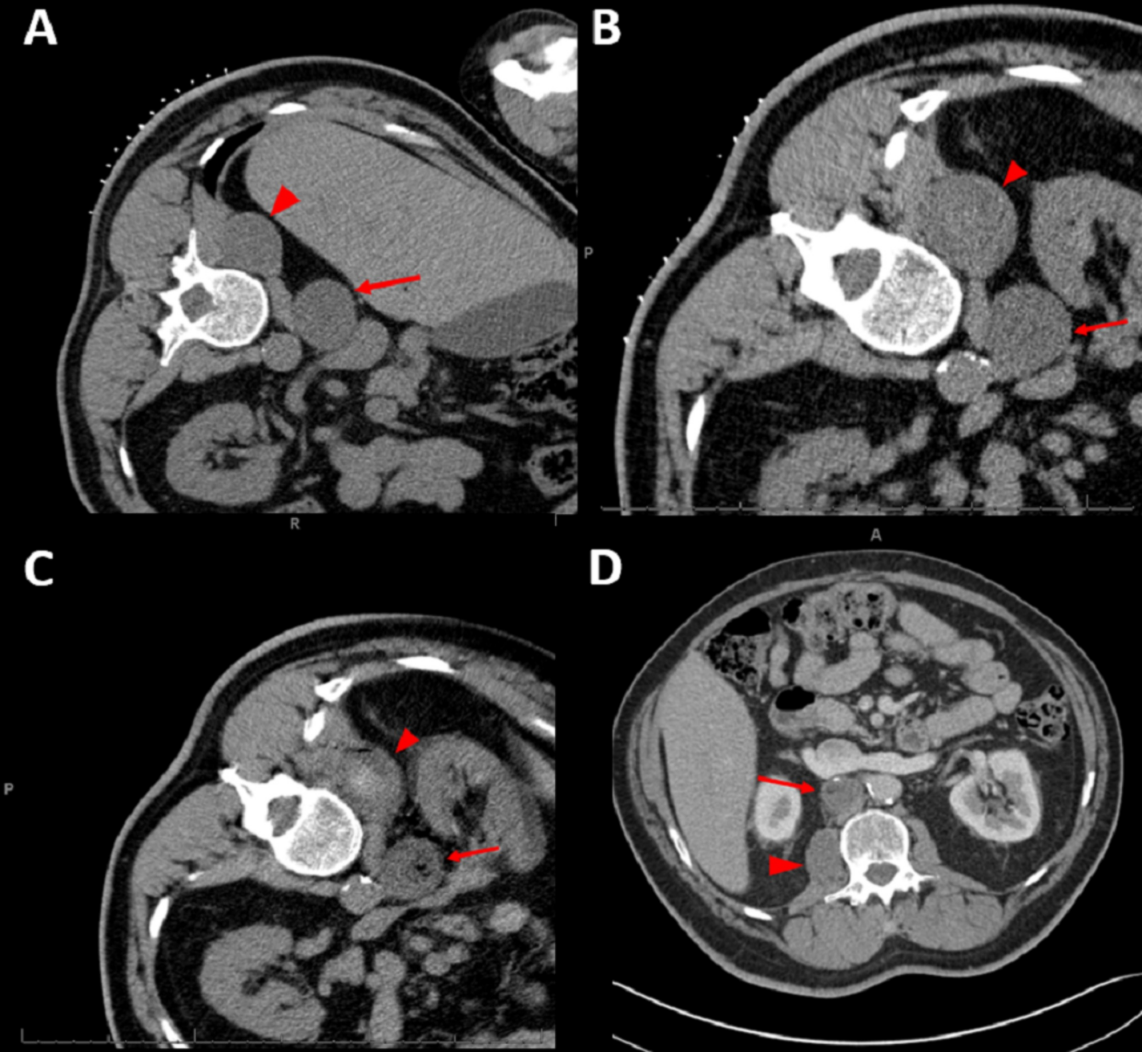
Pre and post-ablation images of the two retroperitoneal neurofibromas 1A. The CT scan shows the two lesions at the time of presentation/biopsy. 1B. The CT scan at the time of the RFA three years after presentation. 1C. Immediate post-ablation CT images show the anticipated changes, with a small amount of air within the anterior lesion (arrow) and a hyperdense hemorrhage within the paraspinal lesion (arrowhead). 1D. Four years post-ablation. Both lesions show a sustained decrease in size. (The anterior lesion is denoted with the arrow, and the paraspinal lesion is denoted with the arrowhead) CT: computed tomography; RFA: radiofrequency ablation

The patient is a 49-year-old male with longstanding back and left leg pain resistant to pain management. He developed acute worsening of his left sciatic pain and suffered a fall with a left wrist fracture. He subsequently developed shortness of breath and was seen in the emergency room. A computerized tomography (CT) study revealed two right retroperitoneal masses, a right prevertebral lesion measuring 4.1 x 3.6 x 5.7 cm with anterior displacement of the inferior vena cava (IVC) and a right paraspinal lesion centered in the psoas measuring 4.0 X 3.5 x 6.6 cm (Figure 1A).

**Figure 2:**
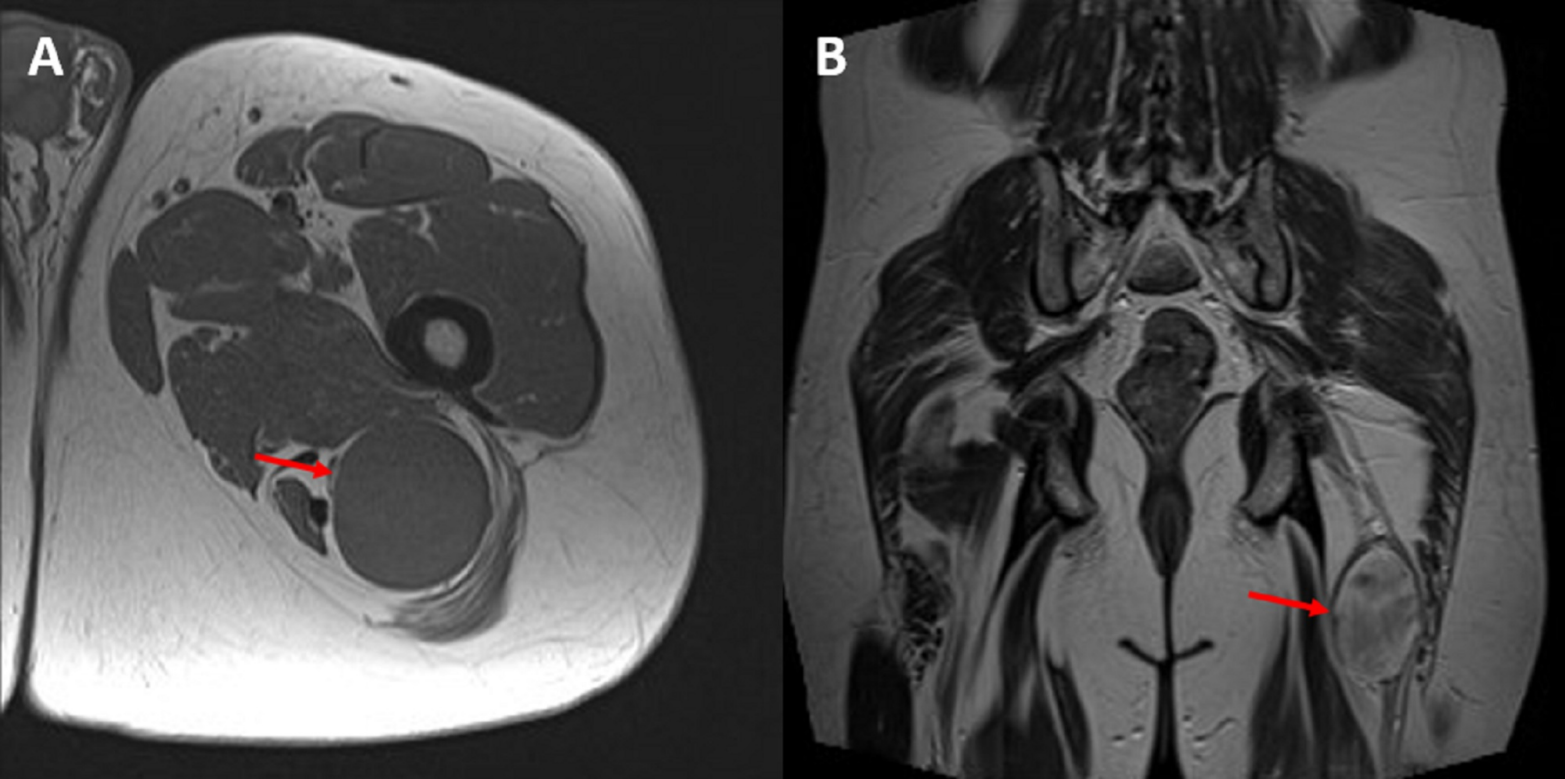
Imaging of sciatic tumor 2A. Axial T1 and 2B. Coronal T2-weighted MR images demonstrating the sciatic nerve tumor (arrow) in the posterior left thigh that was resected uneventfully shortly after the initial presentation with a resolution of the patient’s long-standing left sciatica MR: magnetic resonance

The percutaneous biopsy of these lesions was consistent with a benign nerve sheath tumor. He was sent for neurosurgical management and, during his evaluation, was noted to have an 8 x 5 x 5 cm left sciatic tumor (Figure 2A, Figure 2B).

This was excised uneventfully and found to be a Grade I neurofibroma. His chronic left sciatica resolved although his back discomfort persisted.

Despite the multiple neurofibromas, the patient did not meet the criteria for NF1. He had a family history of multiple melanomas and other malignancies and was sent for genetic evaluation. He was found to have a large, contiguous genetic deletion of chromosome 9p21.3 extending beyond the cyclin-dependent kinase inhibitor 2A (CDKN2A) gene and spanning approximately 25 genes [5].

His medical comorbidities included uncontrolled insulin-dependent diabetes mellitus with a HgA1C of 11.0, complicated by neuropathy and renal insufficiency, a cerebrovascular disease with two prior cerebral vascular accidents (CVA), and mild residual left hemiparesis, a peripheral vascular disease involving iliac stenting, tobacco abuse, and obesity.

The retroperitoneal masses were followed with imaging and were stable for three years. He then developed progressive back and radicular abdominal pain. Imaging revealed the growth of the right prevertebral lesion to 4.4 x 4.0 x 6.2 cm and the growth of the right paraspinal lesion to 4.7 x 4.0 x 7.5 cm (Figure 1A). Neither tumor had changes concerning for malignant degeneration. Given the tumor locations, the progression of the patient’s comorbidities, and his predisposition to develop malignancy, he was not felt to be a good candidate for surgical excision or radiosurgery. For these reasons, he was referred to interventional radiology for image-guided RFA. The procedure was performed with the aid of anesthesia. Under CT guidance, a 17G 3-cm burn radius ablation needle (Cool-tip, Covidien, Boulder, CO, USA) was advanced to each of the lesions, with a 12-minute burn cycle performed at each site (Figure 1B, Figure 1C). He had no complications.

At six weeks post-ablation, the right prevertebral lesion had decreased in size to 3.4 x 3.5 x 5.4 cm and the right paraspinal lesion now measured 3.3 x 4.2 x 6.3 cm (figure not shown). Six months after the procedure, he reported his pain had improved. He no longer required methadone for pain control. On the most recent imaging performed four years after the ablation, the anterior prevertebral lesion shows stable regression at 2.9 x 3.3 x 3.5 cm with the posterior paraspinal lesion further decreasing in size to 2.6 x 3.6 x 5.1 cm (Figure 1D). Table 1 shows tumor measurements over time.

**Table 1:** Tumor Measurements Over Time

	3 years prior to treatment (Time of Diagnosis)	Time of Radiofrequency Ablation	6 weeks post-ablation	4 years post-ablation
Anterior spinal lesion	4.1 x 3.6 x 5.7 cm	4.4 x 4.0 x 6.2 cm	3.4 x 3.5 x 5.4 cm	2.9 x 3.3 x 3.5 cm
Posterior paraspinal lesion	4.0 x 3.5 x 6.6 cm	4.7 x 4.0 x 7.5 cm	3.3 x 4.2 x 6.3 cm	2.6 x 3.6 x 5.1 cm

## Discussion

Peripheral nerve sheath tumors (PNSTs) can arise sporadically or in the presence of genetic disorders, such as NF1, NF2, and schwannomatosis, and in association with large genetic deletions involving the CDKN2A gene, as was noted in our patient [2,5]. In the symptomatic, benign PNST treatment regimen, surgical resection has the greatest opportunity for a complete tumor cure and can typically be carried out with a low risk of creating pain or a new neurological deficit [1-2]. Unfortunately, not all patients are surgical candidates. Pre-existing conditions or tumor location may limit a patient’s surgical options for gaining access to the lesion due to the risk of anesthesia or morbidity. Thus, novel therapeutic approaches must be assessed for efficacy in PNST treatment when a patient is not amenable to surgical resection.

In addition to expectant management with serial imaging and surgical resection, current alternative therapeutic options for non-cutaneous PNSTs include radiosurgery, cryoablation, RFA, and microwave ablation. The CDKN2A mutation detected in our patient has been associated with sporadic and radiation-associated malignant PNST [3]. Given the potential for malignant degeneration in NF1 patients and in those with CDKN2A mutations, alternative treatment options other than radiation for tumor control need to be considered. Percutaneous treatment, as used in this case, provides a viable but underutilized option. We reviewed the literature and found prior reports of percutaneous treatment of PNSTs in only seven patients [4,6-9].

Sanchez et al. utilized cryoablation for the local tumor control of a recurrent paraspinal plexiform schwannoma in a three-year-old boy. The patient underwent resection of the tumor and re-resection of a recurrent nodule. He then underwent the cryoablation of two subsequent recurrences with good tumor control at the last follow-up [6]. Three additional patients underwent successful cryoablation of hip region schwannomas [7-8]. One tumor was in a patient with schwannomatosis [8]. The other two patients had sporadic schwannomas diagnosed by biopsy [7]. The latter two patients developed significant post-procedural pain treated with medication and one patient required an iliohypogastric nerve injection upstream from the tumor during the post-procedural period.

Zhao et al. reported on two patients with retroperitoneal nerve sheath tumors treated with RFA. The first patient had a recurrent malignant retroperitoneal schwannoma treated initially with transarterial chemoablation and radiation. As an adjunct, the patient underwent three RFA treatments over a six-month period. The tumor then decreased in size and was stable for over five years. A second patient had a benign, non-operated retroperitoneal schwannoma that also decreased in size and was stable for 27 months [4]. Yan et al. [9] utilized microwave ablation as a salvage procedure in a patient with a large abdominal malignant peripheral nerve sheath tumor (MPNST) that recurred within two months of surgical resection. Although imaging studies immediately post-ablation showed necrosis in the tumor, the patient died within three months of the procedure [9]. This was the only reported case of treatment failure.

Ours is the first case of the percutaneous treatment of retroperitoneal neurofibromas. The patient's tumors decreased in size following treatment and have remained stable over a four-year follow-up period. One week after the RFA treatment, the patient’s pain began to improve and he was able to eventually stop his methadone treatment. The case details of all patients, including our case, with the percutaneous treatment of PNSTs are summarized in Table 2.

**Table 2:** Case Details Cool-tip radiofrequency ablation needle (Cool-tip, Covidien, Boulder, CO, USA); PerCryo cryoablation probe (Healthtronics, Austin, Texas, USA); IceSphere CryoProbe (Verruca-Freeze, Nashville, TN, USA)

Study	Tumor type	Symptoms	Catheter used	Treatment	Outcome
Radiofrequency Ablation					
Zhao et al. 2012 [4]	Relapsed retroperitoneal malignant schwannoma	Epigastric and left abdominal pain	17 gauge 1.5-mm diameter with a 3-cm active tip	12 minutes of ablation time at each tumor site for a total of four hours during the first treatment and 30 minutes for each of the two supplemental treatments	Imaging demonstrated tumor necrosis and the tumor was stable for over five years following RFA treatment
Zhao et al. 2012 [4]	Retroperitoneal schwannoma	Abdominal pain	17 gauge 1.5-mm diameter with a 3-cm active tip	12 minutes of ablation time at each tumor site for a total of 50 minutes	Tumor decreased in size and was stable for 27 months
Current report	Two retroperitoneal neurofibromas	Severe back and radicular abdominal pain	17-gauge 3-cm burn radius Cool-tip radiofrequency ablation needle	12-minute burn cycle was performed for each mass	Both tumors significantly decreased and have remained stable for over four years
Microwave Ablation					
Yan et al. 2012 [9]	Retroperitoneal malignant peripheral nerve sheath tumor (MPNST)	Abdominal pain	Unknown	55-70 Watts for 75 minutes	Immediate post-treatment imaging showed necrosis in the tumor but eight days later, a CT scan demonstrated an even larger lesion and the patient rapidly deteriorated
Cryoablation					
Sanchez et al. 2017 [6]	Recurrent plexiform schwannoma	Acute spinal cord compression	Endocare cryoprobes	Six-minute freeze cycle followed by an eight-minute thaw followed by a 3-minute freeze cycle	A significant decrease in the tumor size and enhancement was seen at the 70month follow-up
Martell et al. 2016 [8]	Schwannoma in patient with Schwannomatosis	Pain of the lower extremity	17-gauge PerCryo cryoablation probe	Five-minute freeze cycle followed by probe thaw, followed by a five-minute freeze cycle	Resolution of her pain and no new sensory deficits at the six-month follow-up
Mavrovi et al. 2016 [7]	Two cases of peripheral schwannomas	Right buttock pain	IceSphere cryoprobe	Both patients were treated with cycles of freeze, thaw, and freeze, for 10 minutes each.	No recurrence of the lesion based on imaging at the six-month follow-up

The primary concern regarding the percutaneous treatment of benign PNSTs is that the patient might develop neurological deficits or pain as a result of the treatment, either due to direct nerve fascicle injury or secondary to hematoma formation. Significant pain was noted in two of the patients after cryoablation, as described above, although the pain did improve after approximately one week [7]. No other significant procedure-related complications were noted, and with the exception of the aggressive malignant peripheral nerve sheath tumor (MPNST), the percutaneous treatments were successful. Schwannomas typically have only a single non-functioning fascicle running into the tumor and are likely at a lower risk for a neurologic deficit with percutaneous ablative treatment modalities than neurofibromas that may have multiple nerve fascicles coursing through portions of the tumor [1,10]. This may explain why none of the prior reports involved the treatment of neurofibromas. In our patient, the location of the lesions made it unlikely that he would develop a new neurological deficit.

For non-neural malignant tumors, the treatment goal is complete ablation with a treatment margin [4]. For benign PNSTs within major peripheral nerves, the treatment objective should be tumor control and not complete ablation since most normal fibers travel in the capsule or pseudocapsule of the lesion [1]. Theoretically, limiting the therapeutic circumference by intentionally leaving a small, untreated margin and mapping the normal fiber course will minimize treatment-related pain and neurological deficits. Neuromonitoring and the utilization of a newer imaging technology with high-resolution ultrasound and diffusion tensor tractography mapping of tumor-associated nerve fascicles should aid in the planning of needle placement and treatment corridors [10].

A tissue diagnosis should be obtained prior to percutaneous treatment since the management of malignant PNSTs is much more aggressive than that of benign lesions. This is particularly important in patients with NF1 who have a higher risk of harboring malignant PNSTs, have a larger tumor burden, and for whom the decision-making process is often more complex than in a patient with a solitary lesion [3].

If the percutaneous treatment of a nerve tumor fails and patients need to undergo surgical resection for continued growth, pain, or a progressive neurological deficit, an additional concern is that prior percutaneous treatment may make subsequent surgical resection more difficult. In Kline’s series, patients undergoing peripheral nerve tumor resection who had a prior biopsy or partial tumor resection were more likely to develop postoperative pain and neurologic dysfunction [1]. Whether this phenomenon will be true in percutaneously treated tumors remains to be seen, but given the potential, it needs to be carefully considered and discussed with patients prior to treatment.

## Conclusions

In a limited number of cases, percutaneous techniques have been used successfully in the management of PNSTs. Further studies in a larger series of patients with PNSTs need to be performed to fully assess the effectiveness of these therapeutic modalities. The exact indications remain to be defined but given the risk of the development of neurological deficits, neurosurgical involvement is critical for safely advancing this field. In the hands of appropriate multidisciplinary teams, these percutaneous techniques have the potential to become powerful adjunctive tools in the treatment of patients with PNSTs.
